# Occupational group, educational level, marital status and deleterious habits among individuals with maxillofacial fractures: retrospective study

**DOI:** 10.4317/medoral.21969

**Published:** 2017-12-24

**Authors:** Diego-Felipe-Silveira Esses, Fábio-Wildson-Gurgel Costa, Carlos-Diego-Lopes Sá, Paulo-Goberlânio-de Barros Silva, Thâmara-Manoela-Marinho Bezerra, Francisco-Samuel-Rodrigues Carvalho, José-Rômulo de Medeiros, Eduardo-Costa-Studart Soares

**Affiliations:** 1 DDS, MSc, Assistant Professor, Division of Oral and Maxillofacial Surgery, University Center Católica de Quixadá, Quixadá, Ceará, Brazil; 2 DDS, MSc, PhD, Adjunct Professor, Division of Oral and Maxillofacial Surgery, Walter Cantídio University Hospital, Federal University of Ceará, Fortaleza, Brazil; 3 DDS, MSc, Division of Oral and Maxillofacial Surgery, Paulo Picanço School of Dentistry, Fortaleza, Ceará, Brazil; 4 DDS, MSc, PhD, Assistant Professor, Division of Oral Pathology. Unichristus University Center, Fortaleza, Ceará, Brazil; 5 DDS, MSc, Postgraduate student, Division of Oral Pathology, Federal University of Ceará, Fortaleza, Brazil; 6 DDS, MSc, Assistant Professor, Division of Oral and Maxillofacial Surgery, University of Fortaleza, Fortaleza, Ceará, Brazil; 7 Full Professor, Division of Oral and Maxillofacial Surgery, Walter Cantídio University Hospital, Federal University of Ceará, Fortaleza, Ceará, Brazil

## Abstract

**Background:**

To investigate the occupational profile, educational level, marital status and deleterious habits to the health of patients with maxillofacial fractures of a population of northeastern Brazil.

**Material and Methods:**

A retrospective study of patients records admitted to the Division of Oral and Maxillofacial Surgery at the Walter Cantídio University Hospital (Fortaleza, Brazil) who sustained maxillofacial fractures was conducted in the period between 2006 and 2015.

**Results:**

A total of 338 patients rendered 355 fractures. Males were the most affected (*p*<0.001), with prevalence in the third decade of life (*p*<0.001). There was a predominance of motorcycle accidents (*p*<0.001), home workers (*p*<0.001), low educational status (*p* = 0.032), and no cigarette use (*p*<0.001) or alcohol (*p* = 0.023). Fractures of the zygomatic-orbital complex were the most prevalent in the sample (*p*<0.001).

**Conclusions:**

The sociodemographic profile exerted a significant influence on the epidemiological profile of maxillofacial fractures in a Brazilian population during the study period.

** Key words:**Epidemiological studies, trauma, facial bones.

## Introduction

Maxillofacial trauma is a devastating aggression found in large trauma centers, which due to the emotional consequences and the possibility of deformity, often requires multidisciplinary care, with professionals such as otorhinolaryngologists, ophthalmologists, plastic surgeons, neurosurgeons and bucomaxillofacial surgeons, and it has significant economic impact on the health system (1). Trauma, in general, mainly affect urban men, usually young, with maxillofacial lesions being present in a significant number of these patients. These, when present, are most frequently associated with severe morbidity, loss of function, substantial financial cost and deformity (2).

Regarding causes, several studies clearly suggest that maxillofacial lesions vary from country to country and even within the same nation, as different regions suggest different personal behavior patterns. As an example, legislative changes and preventive / surveillance measures involving seat belts and airbag use, as well as the reduction of drinking and motor vehicle driving are directly related to the decrease in the incidence of facial injuries in some developed countries, which differs from interpersonal violence and falls that have emerged as the predominant mechanisms of facial trauma (3). Facial traumatism presents a heterogeneous etiology, and the predominance of a causative factor is related to some characteristics of the studied population, such as age, gender, social, local, urban and residential classification (4). Studies have shown that men are usually more exposed to trauma because they represent the largest number of motor vehicle drivers, because they practice more physical contact sports, besides ingesting more alcohol and other drugs (5). There are also reports in the literature that some population characteristics, such as living in rural and urban environments and socioeconomic or educational levels, influence the etiopathogenesis and severity of facial traumas (6,7).

According to Chrcanovic (8), several risk factors have been related to face trauma, totaling ten, of which can be highlighted: age, sex, geographical region and its cultural aspects, socioeconomic status and climatic influence, alcohol and drug use, compliance of traffic legislation, domestic violence, osteoporosis and the etiology of maxillofacial trauma; the author also stresses the importance of prevention and intervention programs aimed at reducing the incidence of maxillofacial fractures.

The health-disease relation must be understood as a process related with other social determinants which structure the urban space in an environment permeated by social inequalities (9). In this context, the social position of an individual is characterized by the combination of several aspects, such as educational level, occupation and marital status, where each one of these elements, individually or in combination, can exert positive or negative influences on the health conditions. It is observed that, independently of the social marker and health indicator used, there is a universal tendency of individuals in better social positions to experience better health conditions (9).

The epidemiological profile of trauma in the maxillofacial complex can be a reflection of problems related to health inequalities between groups, which, according to Mackenbach *et al.* (10), are characterized by their socioeconomic status (measured, for example, by educational level and type of work) and represent one of the main challenges of global public health. Several epidemiological studies have been published in the sense of confronting the socioeconomic condition of the patients with the involvement of maxillofacial fractures. According to Montovani *et al.* (11), regarding occupancy, there is a higher incidence of facial traumas in students and masons. da Nóbrega *et al.* (12) outlined the epidemiological profile of 884 medico-legal and social records of woman victims of physical aggression from a Center of Forensic Medicine and Dentistry in a metropolitan area in northeastern Brazil. These authors showed a higher prevalence of maxillofacial trauma among single woman (median age of 27 years) with low schooling, and living in an urban area.

In developing African countries, the increase in revenues from the sale of petroleum has directly affected the incidence of face fractures due to the increase in road traffic volume and the social problem of illiteracy; deterioration of infrastructure, such as roads in poor conditions; increased imports of used vehicles; driving under the influence of alcohol; non-compliance with traffic legislation; and failure to wear seat belts and helmets (13).

Therefore, the objective of the present study was to investigate the epidemiological profile, occupation, educational level, matrimonial status and deleterious health habits among individuals with maxillofacial fractures coming from a tertiary hospital located in the northeast region of Brazil, as well as to determine if such variables can be considered as potential risk factors for occurrence of maxillomandibular fractures.

## Material and Methods

A retrospective cross-sectional study was carried out based on data obtained from all medical records of patients attended by the Division of Oral and Maxillofacial Surgery at the Walter Cantídio University Hospital (Fortaleza, Ceará, Brazil) during the period of November 14, 2006 to June 30, 2015. This public institution is responsible for the tertiary care of patients from the capital and the interior of Ceará, referenced through a municipal and state regulation system, as it does not offer emergency or clinical trauma services. The hospital service where the patients were treated acts as a state reference in the areas of bucomaxillofacial trauma, dental-skeletal deformity, pathology, bone reconstruction, temporomandibular joint surgery and oral surgery.

The sample consisted of medical records of the patients affected by maxillofacial trauma who were surgically treated during the described period. The present study was approved by the Research Ethics Committee of the Walter Cantídio University Hospital (CAAE No. 42744915.6.0000.5045).

Data were collected on sex, age, origin, year of trauma, number of fractures, anatomical location (zygomatic-orbital complex, mandible, maxilla, nasal bones, naso-orbito-etmoidal and frontal), and presence / absence of fracture comminution. In addition, the following occupational groups were adopted according to studies by Flor *et al.* (9) and Consuegra-Sánchez *et al.* (14): household workers, unqualified activities, qualified activities and academic activities. The category “home workers” was represented by jobs that do not require specific skills or knowledge, such as household chores, no declared employment, and retirees. Unqualified activities included rural workers, rural owners without employees, and unskilled manual labor. Qualified activities included skilled hand labor, manual labor supervisors, self-employed without employees, self-employed with employees, and routine manual work on sales and services. Academic activities included professionals and low-level administrators and professionals and senior managers. The educational level was divided into three modified categories according to Haas *et al.* (15): low (unspecified, no schooling, basic education or equivalent), medium (secondary level or equivalent), and high (university studies or equivalent). Regarding marital status, individuals were categorized as “married” and “nonmarital” (single, divorced, and widowed). The deleterious habits included alcohol and cigarettes. The data was tabulated in Microsoft Excel and exported to the Statistical Packing for Social Sciences software version 17.0 for Windows, in which the analyzes were performed with a confidence index of 95%. The chi-square test was used for bivariate analysis and exposed to its odds ratio with their respective 95% confidence intervals. The variables were submitted to analysis by multinomial logistic regression model and exposed to adjusted odds ratios obtained in the regression model with their respective 95% confidence intervals.

## Results

The sample consisted of 338 patients, with prevalence of males (n = 278; *p* <0.001), which totaled 355 fractures (number of fractures: number of patients ratio of 1.05). The mean age of all patients was 31.3 ± 12.9 years, with statistical significance for the third decade of life (*p* <0.001). There was no difference in the number of interior and capital patients (*p* = 0.644) ( Table 1).

**Table 1:**
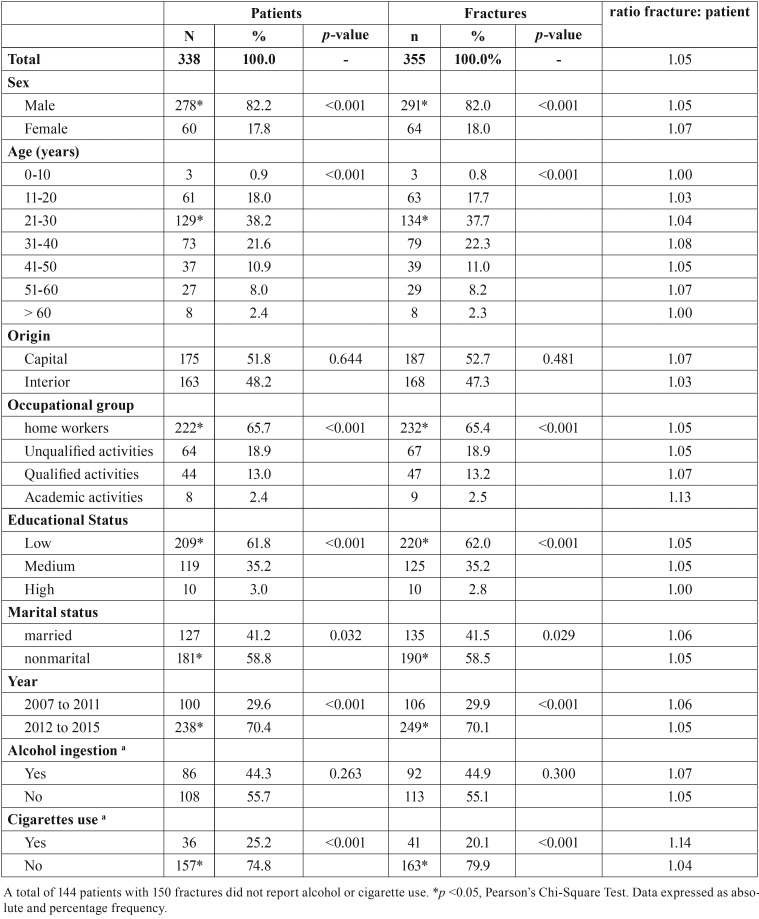
Characterization of patients with maxillofacial fractures, between 2006 and 2015.

With regard to etiology, this work registered a total of 11 motor vehicle accidents (3.3%) and 155 motorcycle accidents (45.9%). Among these, the number of motorcycle accidents was statistically significant (*p* <0.001). With regard to non-automobile accidents (n = 172, 54.1%), there were 21 cyclists (6.2%), 36 sports (10.7%), 6 work accidents (1.8%), 68 physical aggressions (20.1%), 17 fall from own height (5.0%), 4 pathological fractures (1.2%), 3 fractures associated with the extraction of third molars (0.9%), 7 due to firearms lesions (2.1%) and other causes (3%).

Regarding the occupation, there was a significant number of workers in the household (n = 222, 65.7%) in relation to patients with non-qualified (n = 64, 18.9%), qualified (n = 44, 13.0%) or academic activities (n = 8; 2.4%) (*p* <0.001). The most prevalent educational status was the low (n = 209, 61.8%), in relation to mean (n = 119, 35.2%) and high (n = 10, 3.0%) status, and the most prevalent marital status was represented by patients without marital bond (n = 181, 58.8%), compared to patients with marital bond (married n = 121) (*p* = 0.032;  Table 1).

There were 14 (4.1%) hospital admissions in 2007, 9 (2.7%) in 2008, 28 (8.3%) in 2009, 16 (4.7%) in 2010, 33 (9.8%), In 2011, 76 (22.5%) in 2012, 62 (18.3%) in 2013, 57 (16.9%) in 2014 and 43 (12.7%) in 2015, with a linear growth trend (*p*= 0.019, r = 0.753). The highest number of consultations occurred between 2012 and 2015, when compared to other years (*p* <0.001;  Table 1). Most patients did not report alcohol use (*p* = 0.023) and did not report cigarette use (*p* <0.001;  Table 1).

Regarding the fracture type ( Table 2), the sample consisted mostly of fractures of the zygomatic-orbital complex (n = 152), followed by mandible fractures (n = 144), bones of the nose (n= 49), maxilla (n = 7), naso-orbito-ethmoidal (n = 2), and frontal bone (n = 1). Mandibular fractures were significantly prevalent in household workers (*p* <0.001), with low educational status (*p* <0.001), without marital bonding (p = 0.005) and who did not report alcohol (*p* <0.001) or cigarette use (*p* <0.001). Fractures located in the zygomatic-orbital complex were significant in household workers (*p* <0.001), with low educational status (*p* <0.001). Nose bones fractures were significant in household workers (*p* <0.001), with low educational status (*p* <0.001), absence of alcohol use (*p* = 0.001) and cigarette smoking (*p* <0.001).

**Table 2:**
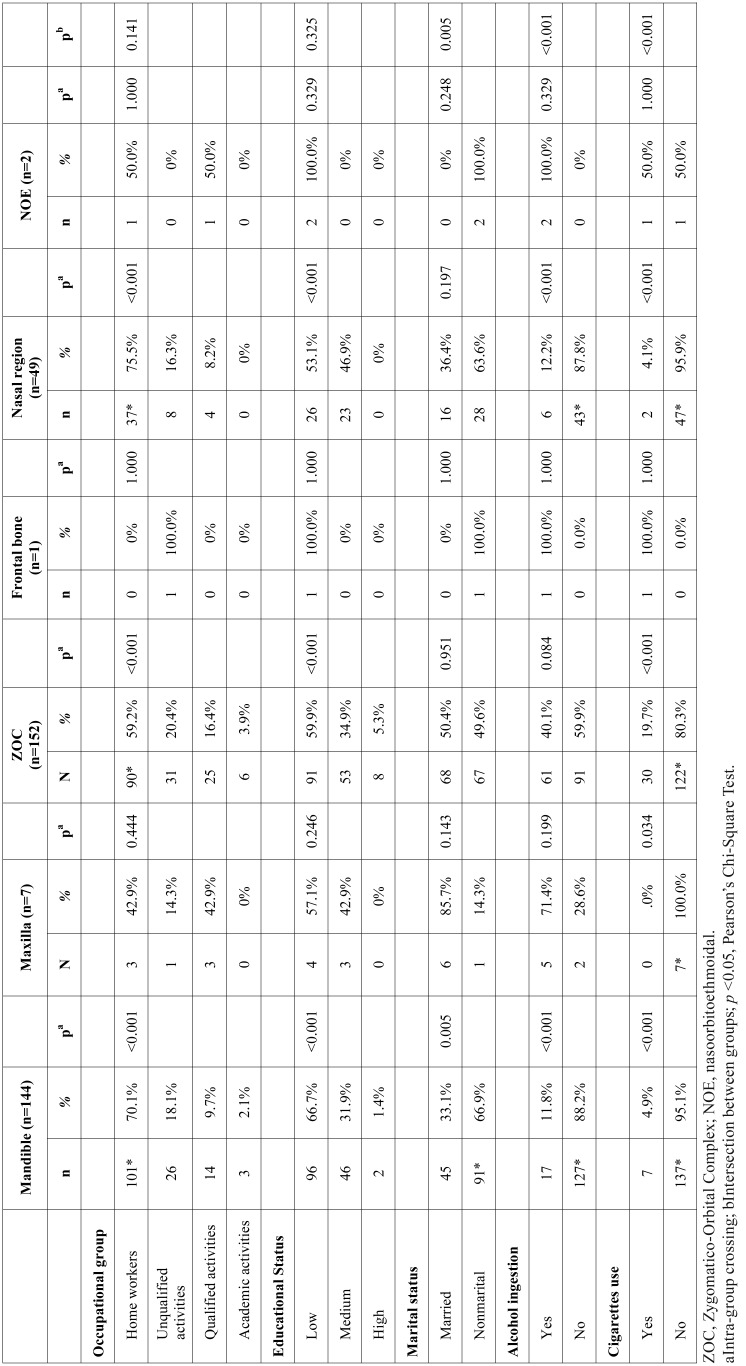
Sociodemographic profile regarding to maxillofacial fracture type (period between 2006 and 2015).

Sociodemographic profile: bi and multivariate analyzes

Regarding socio-demographic characteristics, sex did not show a significant association with educational status (*p* = 0.201) or matrimonial status (*p* = 0.883), as well as with cigarette smoking (*p* = 0.135). Males were more associated with unqualified activities (*p* <0.001) in bi and multivariate analyzes. On the contrary, unqualified activities were 0.1 times less found in females, and a higher prevalence of household workers was found in such individuals. Only males were significantly related to alcohol use (*p* = 0.049;  Table 3). Age was classified in groups (0-30, 31-60,> 60 years) and it had no influence on the occupational group (*p* = 0.672), as well as on educational status (*p* = 0,269). Patients with matrimonial bonds were 5.3 (95% CI 3.3-8.7) times more frequent in the age group between 31 and 60 years, and 22.4 (95% CI 2.7 - 187.8) times More than 60 years of age (*p* <0.001). In addition, alcohol (*p* = 0.594) and cigarette (*p* = 0.713) were not influenced by age when evaluated at 30-year intervals. Reduction in up to ten times of fractures of angle (95% CI 0.0 - 0.9) or mandible condyle (CI 95% 0.0 - 0.3) and fracture trace comminution (CI 95% 0, 0 - 0.6) was observed in household workers and patients with unskilled activities ( Table 4).

**Table 3:**
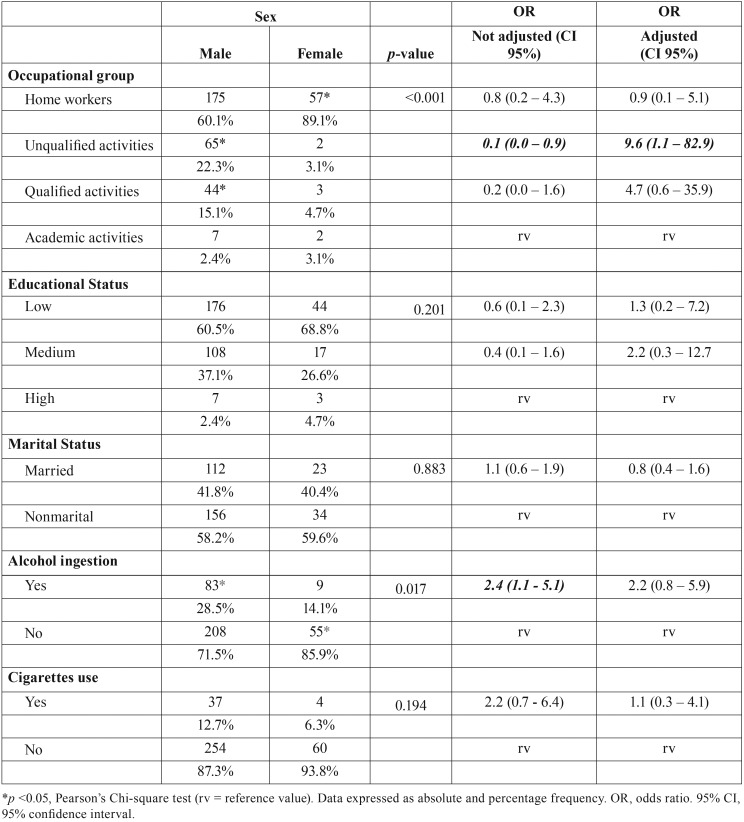
Sex influence on the sociodemographic profile of patients with maxillofacial fractures between 2006 and 2015.

**Table 4:**
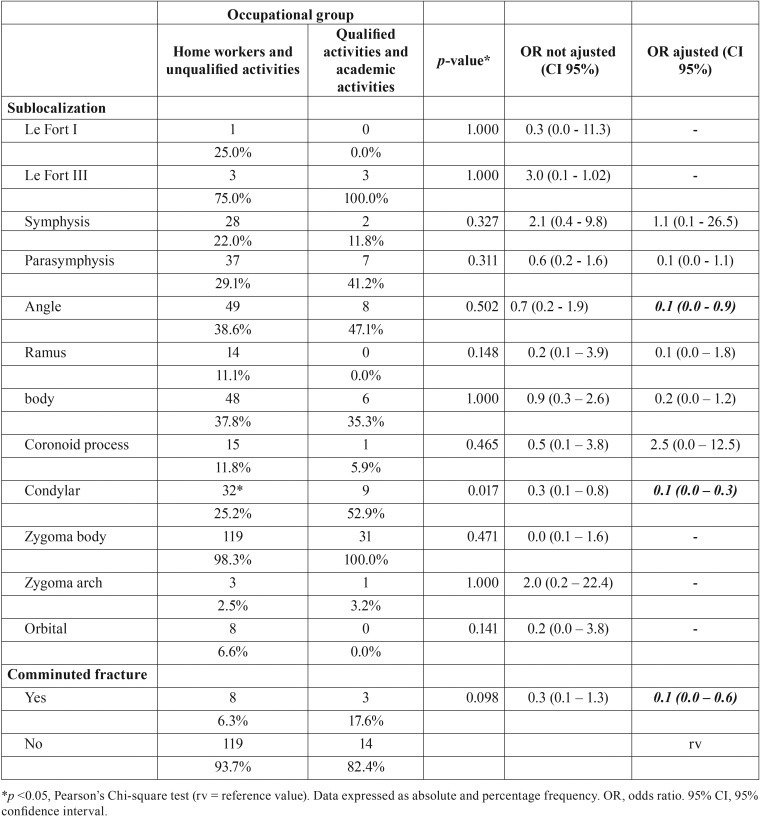
Influence of the occupational group on anatomical sublocalization and comminution of the fracture trait in patients with maxillofacial fractures, between 2006 and 2015.

## Discussion

According to data from the international literature, traumas are among the main causes of morbidity and mortality. The number of worldwide deaths due to the consequences of trauma in 2015 was estimated at 4.7 million (16). Trauma has been considered the leading cause of death in individuals aged 1-44 years and the main cause of lost productivity in a specific population (17), which agrees with the data obtained in this study, since it was observed that the highest prevalence of facial trauma was in the third decade of life. This information highlights the importance of identifying risk factors and use of preventive measures for injuries, as it would reduce the number of deaths, as well as disability or withdrawal from work or student activities, due to trauma (18).

Currently, the association of alcohol, drugs, vehicle management and urban violence increase is increasingly present in the etiology of facial trauma, even increasing its complexity. Thus, there is a need to know the cause, severity and time distribution to set priorities for effective treatment and prevention of these injuries, which is related to the identification of possible direct or indirect risk factors for facial trauma (19).

According to data from an analysis regarding the trend and impact of mortality due to external causes in Mexico, traffic accidents mortality decreased over the years (2000-2013); in addition, the main impact in mortality rates due to external causes was observed in adolescent and adult males aged between 15 and 49 years (20). In the context of the present study, which obtained a higher prevalence of etiology for traffic accidents (motorcycles and motor vehicles), is still necessary to reinforce preventive measures and continuous surveillance.

Road traffic injuries remain as the leading cause of death and disability among young individuals aged between 15 and 29 years, including high-income countries (21). These data are important in the evaluation of possible risk factors for oral and maxillofacial trauma, since in our study we observed a higher prevalence in patients in the third decade of life, and most of them victims of auto accidents.

In a study published by Farneze *et al.* (22) in 2016 that described maxilomandibular trauma of patients at a reference center in oral and maxillofacial service, the mean age was 33.7 years old, with men accounting for 81% of the cases. The main etiology was injuries related to traffic accidents (47%), especially motorcycle accidents. The most fractured bone was the mandible (54%) followed by the zygomatic bone (41%). These findings agree the results obtained in the present study, since it was observed a higher prevalence for males in the third decade of life, especially automobile accidents-related trauma, and the mandible as the most affected bone.

It is important to note that in some countries, over the years, there have been changes in the etiology of facial trauma, with interpersonal violence becoming the leader of the statistics (23). This has been attributed to traffic local legislations, which aim mainly to control speed and alcohol ingestion, ordering the use of seatbelts and crash helmets, the safer design and use of roads and vehicles (17). However, in our study, we still observed a higher prevalence of traffic accidents as etiology of facial trauma. This shows there is a need for greater investment in governmental strategies in order to prevent traffic accidents, especially in patients with low socio-demographic status, since this segment of the population is being more affected by this disease.

Despite the greater number of patients coming from the capital, the absence of a significant prevalence in the patients’ origin can be explained by the importance of the hospital analyzed for the state of Ceará, being a reference in tertiary care to trauma in the state, even though it does not attend traumatic emergency, which could directly affect these data in our study, as this could lead to a greater origin of patients coming from the capital, since the emergency demand, often, does not allow great displacement of the trauma patient.

The social position of an individual is defined by the combination of several aspects, among them, income, education, occupation and lifestyle. Each of these elements, individually or in combination, may exert positive or negative influences on health conditions. Studies show that, regardless of the social marker and health indicator used, there is a universal tendency of those in better social positions to experience better health conditions and quality of life. In addition, they affirm that measuring the health status of populations allows us to define levels of comparison between groups, to detect inequities in health conditions, different pathologies, geographic areas, social conditions, economic conditions, or related to gender and age (15).

The face, as well as the buccal cavity, is susceptible to the most diverse aggressions, and it is important to emphasize that, among these, facial traumatisms, especially fractures, play a prominent role in emergency care around the world. The manifestations of facial fractures can occur in individuals, varying according to gender, age and race, with some fractures more frequent in a particular population group (18).

Although income is associated to the social class, mainly in the economic scope, the two terms cannot be considered synonymous. Authors explain that the occupation has a special highlight among the attributed criteria and it is a powerful indicator of the individual’s position in the social space (24).

Other authors argue that the allocation of people in different social positions results in an unequal distribution of goods, services, living conditions, social advantages and disadvantages (25,26). This separation of occupational / social produces a set of determinations that generate characteristic risks or potentialities, manifested in the form of a health and quality of life (QoL) profile that differs between these groups populations. Studies have already warned of the strong relationship between poor QoL/health and the fact that it is out of the job market (26,27).

In the last decades, numerous studies have analyzed the incidence of bone fractures in women and men. In this context, Zhou *et al.* (28). carried out a retrospective study to investigate differences in incidence, age distribution, etiology, fracture pattern, associated injuries and occupation distribution among women and men, justifying that they could provide a guide for the conception of prevention and treatment programs. They concluded that the demographic characteristics of maxillofacial fractures in female patients differed considerably from those in male patients, since there were 1,131 patients (881 males and 250 females) had a male-female ratio of 3.5:1 (28). Brown and Cowpe (29) observed that, regardless of what prevails in the socioeconomic and cultural conditions, people in the third decade will have a higher prevalence in studies of facial trauma. However, some results differ significantly from the findings in this study, due to local differences. In a study done in Al-Ain, United Arab Emirates (UAE), the highest incidence of cranio-maxillofacial lesions was found at 16 to 20 years of age. This difference can be explained by the fact that Al-Ain is smaller than other emirates, with less control of traffic and highways, thus less police patrol, with many young adolescents driving unlicensed vehicles (30).

The low occurrence of facial fracture in the elderly and children, 0.9% and 2.4%, respectively, in this study was also verified by other authors, who justified such findings for the attention of family members, longer stay in the home and childhood care, in addition to the characteristics of the third age, such as little social and sports activity, leaving home less and, when they do, being accompanied (31).

The results found in the present study corroborate the findings of other researchers when affirming that men from 20 to 39 years of age present a greater number of maxillofacial injuries (32). We believe this finding maybe is associated with the fact that patients of this age represent a group with intense social interaction, participate in dangerous exercises and sports, drive motor vehicles without safety measures, and are more involved in situations of interpersonal violence, making them the most susceptible group. Most of the patients in this study did not report chronic use of alcohol (55.7%), but some authors have written about this fact, since they affirm that among the factors favoring a greater involvement of men by facial fractures, such as accidents at work and Lack of care in traffic, alcohol can be determinant for maxillofacial trauma (33,34).

When comparing the work of Zhou *et al.* (28). with this study, regarding the origin and occupational group involved in facial trauma, the first one observed a higher incidence in the unemployed (21.4%), followed by workers (19.9%), peasants (15.9%) and students (14.8%). On the other hand, the epidemiological data of this study show a higher incidence of cases coming from the capital (51.8%); regarding the occupational group, there is a predilection for domestic workers (65.75%), in which we include the unemployed, followed by unqualified activities, which corresponded to 18.9% of the cases. This raises questions about being a public hospital where most of the demand would be of a socio-demographic profile that is financially disadvantaged or of low educational level.

This study evidenced that the educational status is directly related to the lower incidence of facial trauma, since the group composed of individuals with graduation or higher level education presented smaller numbers (10 patients, or 3% of the cases) of maxillofacial fractures, which reflects in the patients` quality of life. In this regard, Magalhães (35) reports that education is linked to social position and reflects different risks in getting sick and dying, since it is related to the consumption of health services and it influences family decisions about feeding, body care and prevention of diseases. Thus, it is to be expected that those who are more educated will report a better quality of physical and mental life.

In a discussion on employability and mental/physical health, it has been discussed that the existence of an employment status represent something significant in the individuals wellbeing and health-related quality of life (36). This reinforces the data found in this study, since the group of household workers, which includes unemployed persons, presented the highest incidence of facial fractures (65.7%), and groups of unskilled, qualified and academic activities assumed 18.9%, 13% and 2.4% respectively. A comparative analysis of schooling, age and health indicators, such as periodontal disease, showed that individuals over 30 years of age and with low educational level are more likely to develop periodontal disease, the latter being 53% (15). This is corroborated, in part, by the data found, since the higher educational status is associated with a lower incidence of facial fractures (3.0%), different from the medium and high, which assume values of 35.2 and 65.8%, respectively. According to Haas *et al.* (15), a comparison between the health indicator periodontal disease and trauma of the maxillofacial region, in the aspect of matrimonial status, is plausible since both act in a contrary way. Among individuals with periodontal disease, there is a higher incidence of a matrimonial bond, assuming between 41.8% and 60.3% of the cases, which differs when assessing the face trauma, in which the highest incidence is in the patients without marriage bond, 58.8%, emphasizing the idea that the patients’ health condition may be directly related to demographic, socioeconomic and educational factors.

## Conclusions

The majority of the patients in the present study were male, in the third decade of life, with admission in 2012, a higher prevalence of fractures of the zygomatic-orbital complex, without comminution of the fractured segments. It was also observed a higher prevalence for domestic workers, low educational status, no marital bond, in which the majority of patients did not report alcohol use, nor did they report smoking.
